# CHANGING NURSING HOME CULTURE: LONG-TERM CARE LEADERSHIP ACADEMY

**DOI:** 10.1093/geroni/igz038.2835

**Published:** 2019-11-08

**Authors:** Teri Round, Diana Sturdevant

**Affiliations:** 1 University of Oklahoma, Fran and Earl Ziegler College of Nursing, Oklahoma City, Oklahoma, United States; 2 University of Oklahoma, Fran and Earl Ziegler College of Nursing, McAlester, Oklahoma, United States

## Abstract

Nursing homes that fostered open communication and teamwork were more likely to change their practices and adopt the “It’s Not OK to Fall” program. This pilot study evaluated the feasibility and acceptance of a team-building approach for developing leadership skills to three groups of coworkers: Administrators/Directors of Nursing, charge nurses, and certified nursing assistants (CNA) employed by nursing homes in Oklahoma. Each coworker group received one day of job specific leadership training, with another one half-day session where all levels engaged in team-building exercises. Participant satisfaction with course content ranged from agree-to-strongly agree. All stated that they could apply the leadership strategies at their facility. Administrators/Directors of Nursing found tools for tracking turnover/retention and strategies for improving staff communication helpful. Charge nurses and certified nursing assistants seldom viewed themselves as leaders, found coworker group communication very fragmented, and felt least knowledgeable about nursing home care best practices.

